# A New Strategy for Identification of Coal Miners With Abnormal Physical Signs Based on EN-mRMR

**DOI:** 10.3389/fbioe.2022.935481

**Published:** 2022-07-11

**Authors:** Mengran Zhou, Kai Bian, Feng Hu, Wenhao Lai

**Affiliations:** School of Electrical and Information Engineering, Anhui University of Science and Technology, Huainan, China

**Keywords:** coal miners, occupational health, accurate identification, machine learning, feature screening, intelligent optimization

## Abstract

Coal miners’ occupational health is a key part of production safety in the coal mine. Accurate identification of abnormal physical signs is the key to preventing occupational diseases and improving miners’ working environment. There are many problems when evaluating the physical health status of miners manually, such as too many sign parameters, low diagnostic efficiency, missed diagnosis, and misdiagnosis. To solve these problems, the machine learning algorithm is used to identify miners with abnormal signs. We proposed a feature screening strategy of integrating elastic net (EN) and Max-Relevance and Min-Redundancy (mRMR) to establish the model to identify abnormal signs and obtain the key physical signs. First, the raw 21 physical signs were expanded to 25 by feature construction technology. Then, the EN was used to delete redundant physical signs. Finally, the mRMR combined with the support vector classification of intelligent optimization algorithm by Gravitational Search Algorithm (GSA-SVC) is applied to further simplify the rest of 12 relatively important physical signs and obtain the optimal model with data of six physical signs. At this time, the accuracy, precision, recall, specificity, G-mean, and MCC of the test set were 97.50%, 97.78%, 97.78%, 97.14%, 0.98, and 0.95. The experimental results show that the proposed strategy improves the model performance with the smallest features and realizes the accurate identification of abnormal coal miners. The conclusion could provide reference evidence for intelligent classification and assessment of occupational health in the early stage.

## Introduction

As most coal mines are buried underground, underground mining is a very important mining method, and the technical difficulty and risk of underground mining are high (Blondeel and Van de Graaf, 2018; Liu et al., 2019). Because the underground environment and equipment of coal mines are restricted, the health status of underground miners cannot be ignored (Xie et al., 2020). With the continuous increase in coal mining depth, the underground geological conditions are complex, the mining conditions are difficult, the working environment is poor (Paul et al., 2020), and the possibility of miners suffering from occupational diseases has also increased significantly (Lu et al., 2020). Therefore, how to accurately identify coal miners with abnormal physical signs and make early judgments on miners’ physical health is an important premise for the prevention and treatment of miners’ occupational diseases (Hanoa et al., 2011; Volobaev et al., 2016).

The possible health hazard factors in the working environment of the coal mine mainly include dust (Perret et al., 2017), chemical poisons (Pone et al., 2007), and harmful physical conditions (Takacs et al., 2015), which may affect the health of coal miners. Various physical signs of the human body are interdependent, and the change of each physical sign will not be carried out independently, which is a comprehensive organism (Zachurzok-Buczyńska et al., 2011). When the basic physical signs of the human body are abnormal, the physical status of the human body must be changed. However, if the physical status of the human body is abnormal, one of the physical sign parameters may not change, and these abnormal parameters will be the precursor of occupational disease (Ackermann, 2004; Zhu et al., 2014). Therefore, an accurate assessment of the health status of the human body was made only by comprehensively analyzing a variety of physical signs (Pucciarelli et al., 2019). In the traditional approaches, the evaluation of miners’ health status is primarily performed by experienced doctors by integrating the signs information based on the physical examination report (Wu et al., 2019a). This method is not only time-consuming and laborious, especially for doctors with insufficient diagnostic experience, but also with a higher chance of misdiagnosis and missed diagnosis because of subjective (Mackenzie Ross, 2016). The doctors may only consider a single disease that is related to a single factor without paying attention to the correlation between diagnostic results and different signs.

In recent years, artificial intelligence algorithms have been applied to the intelligent aided analysis and evaluation of physical examination data, which provides a lot of theoretical basis for health management and disease prevention (Hoogendoorn et al., 2016; Grzywalski et al., 2019; Koshimizu et al., 2020; Yang et al., 2020). For example, Wu et al. (2019b) put forward the learning vector quantization and Fisher-SVM to assess the risk of hypertension in steel workers and achieved a good evaluation effect. Lee et al. (2020) developed the prediction model based on XGBoost to assess the risk of metabolic syndrome with body weight control. Maxwell et al. (2017) used a Deep Neural Networks (DNN) algorithm based on deep learning for multi-label classification and prediction of chronic diseases. Galarraga et al. (2017) combined principal component analysis (PCA) and multiple linear regressions to process physical examination and surgery data for the prediction of postoperative gait in cerebral palsy. However, at present, all the attributes of physical examination data were analyzed by some of these machine learning algorithms without considering the redundancy of attribute parameters. The parameter adjustment of the predictive model based on the deep learning method is complex, a large number of samples need to be iterated a certain number of times to achieve the targeted accuracy, and the efficiency is low. The feature extraction methods including PCA change the raw data structure, and the dimensionality reduction results in features without physical meaning, which is not interpretable.

Elastic net (EN) is a feature screening method based on regulation (Teisseyre, 2017), which can effectively solve the over-fitting problem, reduce the relevant features, and remove them from the model. For example, Kocsmár et al. (2020) used the elastic net feature selection method to improve the parameter sample size ratio and found the influence factor in periampullary adenocarcinomas. Fukushima et al. (2019) proposed an improved elastic net to realize the accurate prediction of treatment response for multiple sclerosis (MS) patients. Bravo-Merodio et al. (2019) combined the Elastic Net with different supervised learning methods to assess the quality of clinical biomarkers. Watts et al. (2021) used the elastic net to select important clinical variables from risk factor data to predict different types of criminal offenses. Max-Relevance and Min-Redundancy (mRMR) is a feature selection method that uses the dependence of features and tags and the correlation between features (Bose et al., 2019). Wei et al. (2020) found useful texture features in CT images of COVID-19 by mRMR technology and the prediction model showed a good predictive performance. Aghaeipoor and Javidi (2020) put forward the algorithm based on the mRMR framework to improve the accuracy of the estimation method for real-world regression datasets. Özyurt (2020) applied a feature selection algorithm based on CNN–mRMR and an extreme learning Machine (ELM) classifier to achieve a higher classification accuracy for white blood cell detection. Bose et al. (2019) chose attributes with scores greater than zero and a linear model for identifying the key information in nursing documentation. There are many applications of mRMR in biomedicine. Zhang et al. (2021) used the mRMR and IFS-SVM to classify the microbiota biomarkers with orthologous gene annotation. Wu et al. (2020) combined mRMR with SVM for classifying the osteoarthritis hip samples and osteoarthritis knee samples evaluated with LOOCV. Cai et al. (2018) adopted the mRMR and the variance inflation factor regression algorithm to identify their interacting schizophrenia genes in brains. Chen et al. (2019) used the mRMR and recurrent neural network to select the discriminate features for classifying widely expressed genes. The gravity search algorithm (GSA) is a new swarm intelligence optimization algorithm based on the law of universal gravitation and the interaction between particles (Mosa, 2019). Han et al. (2015) combined the enhanced GSA algorithm with BP neural network to segment the image. Goswami and Chakraborty (2015) employed GSA and fireworks algorithm (FWA) to optimize the parameters of the Ultrasonic machining (USM) process. Guha et al. (2020) developed a new method called Clustering-based Population in Binary GSA (CPBGSA), which improved the classification performance of the model for UCI datasets.

The present work is focused on the development of an analytical method for identification of coal miners with abnormal physical signs based on EN-mRMR and intelligent optimization. Firstly, the physical examination data of coal miners are collected, and the samples are randomly divided into the training set and the test set. Then, the elastic net is used for the initial screening of raw data to obtain important physical signs. The identification model of coal miners with abnormal signs is established by mRMR combined with the GSA-SVC algorithm. The features of the data from preliminarily selected physical signs are further simplified, and the key feature subset is selected to obtain the optimal identification model. Finally, the conclusion and future work plan of this paper are summarized.

## Materials and Methods

### Collection of Physical Examination Data of Coal Miners

With the assistance from the research platform of the Huaihe Energy Hospital for the Prevention and Treatment of Occupational Disease, Biosensing and Comprehensive Health Laboratory of School of electrical and information engineering, and Key Laboratory of Industrial Dust Control and Occupational Health, Ministry of Education, and Anhui University of Science and Technology. The occupational health data of coal miners in the Huainan mine area in 2020 were taken as the research object, and the data set of physical signs were constructed. These coal miners came from six departments, including electromechanical, transportation, fully mechanized mining, development, drivage, and coal preparation plant. The data set contains 320 samples, which are mainly composed of three parts. The first part is the coal miner’s basic information, including age (AGE), length of service (LS), and toxic length of service (TLS). The second part is the coal miner’s physical parameters, including heart rate (HR), systolic blood pressure (SBP), diastolic blood pressure (DBP), height (HGT), weight (WGT), alanine aminotransferase (ALT), triglyceride (TG), cholesterol (CHOL), glucose (GLU), the predicted value (Pred), measured value, ratio of the measured value to the predicted value (%) of forced vital capacity (FVC), and forced expiratory volume in one second (FEV1) and FEV1/FVC. SBP and DBP are the evaluating indicator of cardiac function. The ALT is the evaluating indicator of liver function. TG and CHOL are the evaluating indicators of blood lipid. The GLU is the evaluating indicator of blood glucose. FVC and FEV1 are the evaluating indicators of pulmonary function. The third part is the final evaluation result, including two examination conclusions: “abnormal physical signs that need further clinical examination” and “normality of the items currently examined”. There are 141 coal miners with normal physical signs and 179 coal miners with abnormal physical signs. The proportion of samples with normal and abnormal physical signs of coal miners is about 1:1.27. There is little difference between the number of coal miners with normal and abnormal physical signs. According to the examination results, the category of coal miners is marked.

The hardware conditions of the computer used in the experiment with the Intel ninth-generation core i7-9700, the 3.0GHz eight-core processor, the NVIDIA RTX2070 graphics card (8GB Video memory), the 16G Kingston memory module, etc. The algorithm simulation runs on the MATLAB R2021a (MathWorks, United States) platform.

### Elastic Net

Elastic network (EN) is a new embedded feature selection method (Zou and Hastie, 2005). Based on the lasso algorithm, which can select more representative feature variables. The EN uses both L1 and L2 as regularization terms, L1 regularization makes the model become sparse, and L2 makes the model parameters closer to zero. When the model parameters are limited or normalized, some parameters can shrink toward zero. The L1 controls the number of features so that a few features are more important and can be used for feature selection. L2 cannot control the number of features, but it can prevent the model from over-fitting to a feature.

The EN combines the characteristics of the L1 regularized model based on the lasso and the L2 regularized model based on the ridge, which not only ensures the sparsity of the model but also inherits the stability of the ridge.

L=Xω+E, (1)

where $X={\left[x_{1},x_{2},\cdots ,x_{m}\right]}^{T}\left(X\in R^{m\times n}\right)$ is the attribute variable, $L={\left[l_{1},l_{2},\cdots ,l_{m}\right]}^{T}\left(L\in R^{m\times 1}\right)$ is the result label, $E\in R^{m\times 1}$ is the random error, and $\omega ={\left[\omega_{1},\omega_{2},\cdots ,\omega_{n}\right]}^{T}\left(\omega \in R^{n\times 1}\right)$ is the regression coefficient vector.

Parameter α can be adjusted according to Eq. 1 to achieve sparse dimensionality reduction of target variables.

$$Q\left(\omega \right)=\arg\min\left\{{\left\|L-X\omega \right\|}^{2}+\lambda_{1}\left|\omega \right|+\lambda_{2}{\left\|\omega \right\|}_{2}\right\},$$ (2)

where $\lambda_{1}$ and $\lambda_{2}$ are penalty coefficients, let $\alpha =\frac{\lambda_{1}}{\lambda_{1}+\lambda_{2}}$ , $\lambda =\lambda_{1}+\lambda_{2}$ , and we obtain the following formula:

$$Q\left(\omega \right)=\arg\min\left\{{\left\|L-X\omega \right\|}^{2}+\lambda \left[\alpha \left|\omega \right|+\left(1-\alpha \right){\left\|\omega \right\|}_{2}\right]\right\}.$$ (3)

In the optimization function Q(ω) , the value of α is strictly between zero and one.

### Max-Relevance and Min-Redundancy

The Max-Relevance and Min-Redundancy (mRMR) is a filter feature selection method based on mutual information (Hanchuan Peng et al., 2005), which can select features according to the maximum statistical dependence criterion, and has the advantages of high speed and robustness. The algorithm minimizes the redundancy of feature subsets, maximizes the correlation between feature subsets and response variables, and finds several features with the greatest correlation and the least redundancy with each other from the feature space.

The mRMR is defined as follows:

$$\max D\left(U,l\right),D=\frac{1}{\left|U\right|}\sum_{\xi_{i}\in U}I\left(\xi_{i};l\right),$$ (4)

$$\min R\left(U\right),R=\frac{1}{{\left|U\right|}^{2}}\sum_{\xi_{i},\xi_{j}\in U}I\left(\xi_{i};\xi_{j}\right),$$ (5)

where U is the feature subset, |U| is the feature number, and l is category labels. $I\left(\xi_{i};l\right)$ is the mutual information between feature i and l . $I\left(\xi_{i};\xi_{j}\right)$ is the mutual information between feature i and feature j . D is the mean value between features and categories in feature subsets U , which reflects the correlation between features and category labels. R is the mutual information value between features, which reflects the degree of redundancy between features.

The criteria of mRMR are as follows:

$$\max\phi \left(D,R\right),\phi =D-R=\frac{1}{\left|U\right|}\sum_{\xi_{i}\in U}I\left(\xi_{i};l\right)-\frac{1}{{\left|U\right|}^{2}}\sum_{\xi_{i},\xi_{j}\in U}I\left(\xi_{i};\xi_{j}\right).$$ (6)

mRMR=maxϕ(D,R) , and the ultimate goal is to find the set U with maximum correlation and minimum redundancy.

### Support Vector Classification of Optimization by Gravitational Search Algorithm

The Gravity Search Algorithm (GSA) is a random heuristic search optimization algorithm (Rashedi et al., 2009). This algorithm is inspired by Newton’s law of gravity and motion in physics. A particle is defined as a solution within the range of the solution set. There is an attraction between different solutions, which is affected by the distance between the mass of the solution and the solution. The value of the evaluation function can be used to describe the mass of the particle. In the range of solution set, one solution will get acceleration due to the attraction of other solutions, and the particle with a better evaluation function will provide more acceleration, and then get a better solution. The support vector classification (SVC) is a supervised machine learning algorithm based on the support vector machine for classification problems (Mustafa et al., 2016). The SVC is suitable for small sample learning and simple calculation. It can not only improve the generalization ability of the learning machine by seeking the minimum structural risk but also avoid the disaster of dimensionality in a sense (Manuel Serra et al., 2007). There are two important parameters in the SVC model, one is the cost and the other is the gamma . The cost is the penalty coefficient, which represents the tolerance to error, and the gamma is the kernel function parameter. GSA is used to search for the optimal cost and gamma parameters to improve the performance of the SVC classifier (MadhuSudana Rao et al., 2018).

The steps for GSA to optimize parameters of SVC are as follows:

Step 1 Determined the search space H , the population size K , and the number of iterations N . Initial the gravitational constant $G^{*}$ , the attenuation coefficient α , the position $Z=\left(z_{1},z_{2},\cdots ,z_{i},\cdots z_{n}\right)$ , and the speed $V=\left(v_{1},v_{2},\cdots ,v_{i},\cdots v_{n}\right)$ of the individual. Randomly generate n particles. The position of the particles corresponds to a set of cost and gamma .

Step 2 Determine the search range and iteration times of parameters cost and gamma in SVC.

Step 3 The data of training set is used as the input of the model, the training sample data is trained by SVC, and the classification error rate is used as the optimization objective function.

Error_rate=1−ACC, (7)

where ACC represents the ratio of samples correctly classified.

Step 4 Put the updated cost and gamma into the SVC model and calculate the fitness value of each individual in the fitness function Fit(t) based on the minimum value of an objective function.

Step 5 Update the universal gravitation G(t) , mass M(t) , minimum fitness values $\min F{\text{it}}_{\text{i}}\left(t\right)$ , and $\max F{\text{it}}_{\text{i}}\left(t\right)$ at time t .

Step 6 Calculate the gravity between individuals $F_{ij}\left(t\right)$ and individual acceleration $a_{i}\left(t\right)$ at time t .

$$F_{ij}\left(t\right)=G\left(t\right)\cdot \frac{M_{i}\left(t\right)\cdot M_{j}\left(t\right)}{d_{ij}+\tau }\cdot \left(z_{i}\left(t\right)-z_{j}\left(t\right)\right),$$ (8)

where $d_{ij}\left(t\right)$ represents the Euclidean distance between individuals i , j , and τ is a minimum positive constant.

$$M\left(t\right)=\frac{1}{\sum_{j=1}^{n}\frac{Fit_{j}\left(t\right)-worst\left(t\right)}{best\left(t\right)-worst\left(t\right)}}\cdot \frac{Fit_{i}\left(t\right)-worst\left(t\right)}{best\left(t\right)-worst\left(t\right)},$$ (9)

where best(t) represents the optimal fitness value. worst(t) represents the worst fitness value.

$$a_{i}\left(t\right)=\frac{\sum_{j\in kbest}F_{ij}\left(t\right)}{M\left(t\right)},$$ (10)

where kbest represents the number of best heavy mass elements, which can create the balance between exploration and exploitation processes. Some miscellaneous particles are filtered out to highlight the influence proportion of better individuals.

Step 7 Update the individual’s position $z_{i}\left(t\right)$ and speed $v_{i}\left(t\right)$ .

$$v_{i}\left(t+1\right)=Random\cdot v_{i}\left(t\right)+a_{i}\left(t\right),$$ (11)

$$z_{i}\left(t+1\right)=z_{i}\left(t\right)+v_{i}\left(t+1\right),$$ (12)

where Random is a random variable in the [0,1] interval.

Step 8 Judge whether the maximum number of iterations is reached, and stop when it is reached, otherwise go back to (2) to continue execution.

Step 9 Return the optimal solution of cost and gamma of SVC model.

Step 10 According to the optimal parameters cost and gamma , the optimal recognition model is obtained. The test set is used to evaluate the performance of GSA-SVC model.

### Feature Construction

Machine learning model learns from training data, so it is very important to construct some features for the related task. Feature construction is achieved by studying the raw data samples combined with the experience of machine learning and professional knowledge in related fields. The existing features are combined or calculated with each other to manually create some new physical significant features, which are useful for model training and have certain engineering significance (Neshatian et al., 2012; Mahanipour and Nezamabadi-Pour, 2019). To better evaluate the performance and identification accuracy of the model, we constructed four new features based on the raw physical signs. These new features can be used as a measure of health, including the body mass index (BMI) (Opel et al., 2015), the pulse pressure (PP) (Gavish and Bursztyn, 2019), the mean arterial pressure (MAP) (Deverdun et al., 2016), and the rate-pressure product (RPP) (Keller-Ross et al., 2014).

The expression of BMI:

$$BMI=\frac{W}{H^{2}},$$ (13)

where W represents the weight (kg) and H represents the height (m).

The expression of PP:

PP=SBP−DBP. (14)

The expression of MAP:

$$MAP=\frac{1}{3}\left(SBP-DBP\right)+DBP.$$ (15)

The expression of RPP:

RPP=HR⋅SBP, (16)

where SBP represents systolic blood pressure, DBP represents diastolic blood pressure and HR represents heart rate.

### Evaluation Indexes

The evaluation of model performance can guide the training process of classifiers and compare the performance of different classifiers. Accuracy is our most common evaluation index and is easy to understand. Generally speaking, the higher the accuracy, the better the performance of the model but the high accuracy does not necessarily indicate the algorithm is good. Especially when the uneven distribution of positive and negative samples leads to fewer data in some categories, it is not comprehensive to evaluate an algorithm model only by the index of accuracy. The confusion matrix is shown in Table 1. Through the confusion matrix, we can intuitively observe the distribution of each category. The confusion matrix includes four elements: TP, FP, FN, and TN. The first letter represents the match situation between the predictive result, the actual result of the sample, and the second letter represents the predictive result of the sample. The accuracy, precision, recall, specificity, geometric mean (G-mean), and Matthew’s correlation coefficient (MCC) are used as quantitative evaluation indexes for the quality of algorithm or model parameters.

**TABLE 1:** Confusion matrix.

	Identification result	
Actual result	Abnormity	Normal
Abnormity	True positive (TP)	False negative (FN)
Normal	False positive (FP)	True negative (TN)

The expression of accuracy:

$$Accuracy=\frac{TN+TP}{TN+FN+FP+TP}\times 100\text{\%}\text{.}$$ (17)

The expression of precision:

$$Precision=\frac{TP}{FP+TP}\times 100\text{\%}\text{.}$$ (18)

The expression of recall:

$$Recall=\frac{TP}{FN+TP}\times 100\text{\%}\text{.}$$ (19)

The expression of specificity:

$$Specificity=\frac{TN}{FP+TN}\times 100\text{\%}\text{.}$$ (20)

G-mean is used to measure the evaluation index of the algorithm for a few types of samples and can evaluate the overall identification performance of two types of samples in an unbalanced data set. The closer the value is to one, the better the identification performance is. The closer the value is to zero, the worse the identification performance is. Its expression is

$$G-mean=\sqrt{\frac{TP}{FN+TP}\cdot \frac{TN}{FP+TN}}.$$ (21)

MCC is an evaluation index to measure the identification performance of the model. The closer the value is to one, the better the identification performance of the model is. The closer the value is to minus one, the worse the identification performance is. Its expression is

$$MCC=\frac{TN\cdot TP-FN\cdot FP}{\sqrt{\left(FP+TP\right)\left(FN+TP\right)\left(FP+TN\right)\left(FN+TN\right)}}.$$ (22)

## Results and Analysis

### Physical Sign Screening of Elastic Net

The physical signs data of coal miners contains 25 variables after feature creation, but the physical health of coal miners only depends on a few unique and useful signs. Therefore, the screening of sign variables can not only reduce the identification cost but also effectively improve the accuracy, which is very important for the actual auxiliary diagnosis and evaluation process. The EN feature screening algorithm is used to select useful physical signs to avoid the adverse impact of redundant physical signs on auxiliary diagnosis results. The data set of coal miners’ physical signs consists of 320 samples. These samples were randomly divided into the training set and the test set according to the ratio of 3:1. The training set contains 240 samples and the test set contains 80 samples.

The training set of data are fed into the EN algorithm. The output is the feature coefficient corresponding to different alpha. The features of the zero coefficient will be deleted. It can be seen from Eq. 3 that the variable screening of EN is related to the two parameters of alpha and lambda in the optimization function. The value range of alpha is (0, 1), and the value of alpha is set to 0.1, 0.2, 0.3, 0.4, 0.5, 0.6, 0.7, 0.8, and 0.9 at intervals of 0.1. Then, a 5-fold cross-validation is adopted based on the cross-validation of the deviation criterion. The results of physical signs screening are shown in Table 2. When alpha is 0.1, the number of deleted physical signs is the least, only the 10th physical sign is deleted, and the sign variable corresponding to the non-zero sparsity coefficient is the feature variable. With the continuous increase of alpha, more and more feature coefficients will become zero, and the number of deleted physical signs is gradually increasing. When the alpha is 0.8, the number of remaining physical signs is the least, a total of 12. When the alpha value is incremented from 0.3 to 0.4 and 0.6 to 0.7, the number of deleted features is larger than others. It can be roughly seen from the statistical information of sign parameters deleted by the different alpha that the LS and the measured values of FEV1/FVC appear the most times, a total of eight times. The HGT appeared seven times in total. The SBP, the BMI, and the predicted values of FVC appeared more times, more than five times in total. The deleted physical signs have little impact on the diagnostic results of coal miners’ health assessment, which belong to redundant or useless information in the data.

**TABLE 2:** Results of physical sign screening.

Alpha	Deleted physical signs	Remaining number
0.1	x_10_	24
0.2	x_2_, x_22_	23
0.3	x_2_, x_10_, x_22_	22
0.4	x_2,_ x_5_, x_10_, x_12_, x_17_, x_22_	19
0.5	x_2_, x_5_, x_10_, x_12_, x_17_, x_20_, x_22_	18
0.6	x_2_, x_5_, x_10_, x_12_, x_17_, x_18_, x_20_, x_21_, x_22_	16
0.7	x_2_, x_4_, x_5_, x_7_, x_10_, x_12_, x_17_, x_18_, x_19_, x_20_, x_21_, x_22_	13
0.8	x_2_, x_4_, x_5_, x_7_, x_10_, x_12_, x_17_, x_18_, x_19_, x_20_, x_21_, x_22_, x_23_	12
0.9	x_2_, x_4_, x_5_, x_7_, x_12_, x_17_, x_18_, x_19_, x_20_, x_21_, x_22_, x_23_	13

**FIGURE 1:**
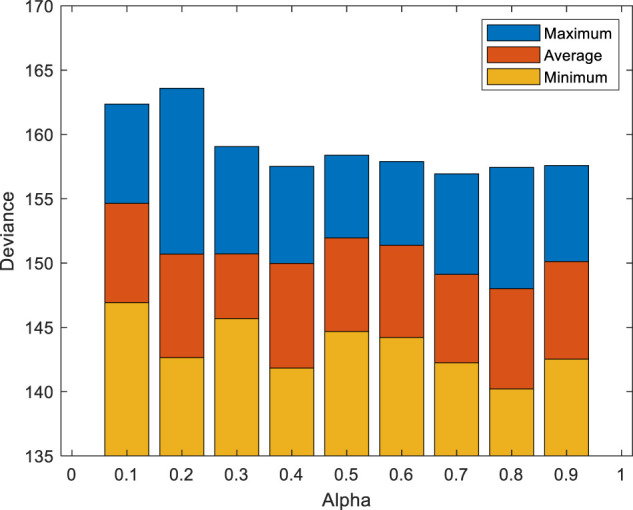
Variation curves of different alpha values. The alpha values range from 0.1 to 0.9, and there are different degrees of differences between the maximum, minimum, and average values. When alpha = 0.8, the minimum and average value of deviance are the minimum values, which are 148.01 and 140.22, respectively.

To further judge the specific impact of different physical signs, each alpha value is substituted into EN to loop 10 times, and the value of alpha is finally determined by the maximum, minimum, and average value of deviation. The variation curves of different alpha values are shown in Figure 1. The alpha values range from 0.1 to 0.9, and there are different degrees of differences between the maximum, minimum, and average values. When alpha = 0.2, the maximum deviance reaches the maximum value of 163.59. When alpha = 0.1, the average value and minimum value of deviance reach the maximum, which are 154.65 and 146.92, respectively. When alpha = 0.8, the minimum and average value of deviance are the minimum values, which are 148.01 and 140.22, respectively. In conclusion, better screening results are obtained through the EN model. (AGE, TLS, DBP, MAP, RPP, WGT, ALT, TG, CHOL, GLU, FEV1%, and FEV1/FVC%) are the final result of selected features.

**FIGURE 2:**
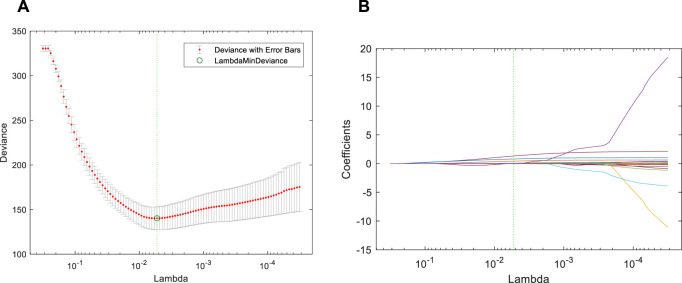
Implementation results of the EN. **(A)** Deviance variation curves. The circle at the green dotted line in the figure refers to the minimum deviance point. The value of minimum *λ* based on the deviance criterion is 0.0053; **(B)** Variation track of coefficient. The position of the green dotted line corresponds to different coefficients.

When the alpha of the cross-validation parameter is 0.8, the deviance variation curves of different regularization coefficients are shown in Figure 2. The circle at the green dotted line in the figure refers to the minimum deviance point. As can be seen from the figure, the value of minimum λ based on the deviance criterion is 0.0053.

### Feature Selection of Max-Relevance and Min-Redundancy

The number of physical signs initially screened by EN is reduced by 13 from 25 which is 48%. This procedure greatly reduces redundant information. However, the mechanism of feature selection by EN is completed by deleting the variable with zero coefficient. There may be redundancy between the deleted 13 individual features. The correlation and redundancy between the physical signs are not fully considered, and the number of physical signs retained is still large. To achieve the accurate and efficient identification of coal miners with abnormal physical signs, the combination algorithm of filter and wrapper is employed for subsequent analysis. Firstly, the mRMR algorithm is used to sort the importance of features, then a specific classifier is used to select features, and the optimal feature subset is simplified according to the evaluation indexes. After the initial selection of EN, the 12 features are selected by the mRMR algorithm.

**FIGURE 3:**
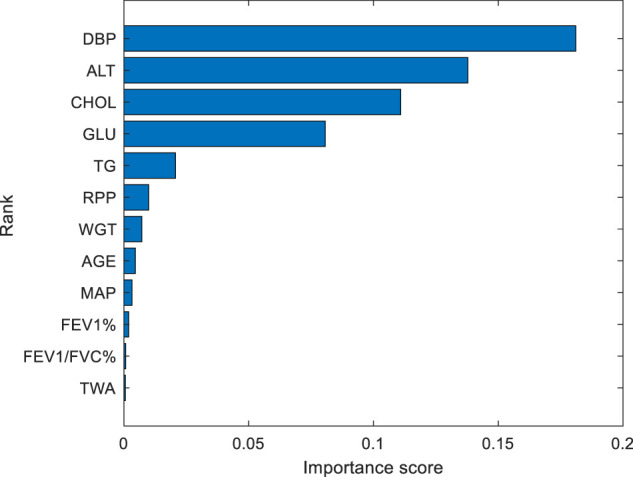
Importance score of different physical signs. From top to bottom, the importance score of 12 physical signs are decreased continuously. The DBP has the highest importance, with a value of 0.18. The TWA is the least important, with a value of 0.0006.

The importance score of different physical signs is shown in Figure 3. According to the value of importance score, the order from large to small is (DBP, ALT, CHOL, GLU, TG, RPP, WGT, AGE, MAP, FEV1%, FEV1/FVC%, and TWA). From top to bottom, the importance score of DBP, ALT, CHOL, GLU, and TG decreased significantly. The decrease in importance score indicates the degree of reliability of feature selection. After that, the decline of the importance scores of the physical signs are not obvious, and they are stable.

### Identification Model for Coal Miners With Abnormal Physical Signs

The initial population number of GSA-SVC is set to 20, the maximum number of iterations is set to 100, the search interval of penalty coefficient cost and kernel function parameter gamma are respectively set to (0,100), and the data are normalized to (0,1). Different kernel functions of SVC will affect the identification performance of the model. The SVC model of linear, polynomial, sigmoid, and radial basis function (RBF) kernel function are, respectively, established for the 12 physical signs data screened by EN. The training set of 12 physical signs data are fed into the GSA-SVC algorithm, and the identification model of coal miners with abnormal physical signs is established. The test set is used to evaluate the performance of GSA-SVC model. The output of the model is the category of the predictive coal miners, accuracy, precision, recall, specificity, G-mean, and MCC of the test set.

**FIGURE 4:**
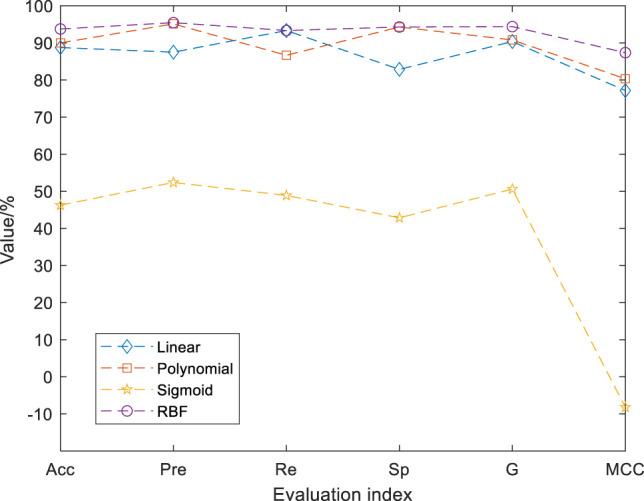
Identification results of different kernel functions. The values of each evaluation index of RBF kernel function are high. The accuracy, precision, recall, specificity, G-mean, and MCC of the test set were 93.75%, 95.45%, 93.33%, 94.29%, 0.94, and 0.87. The values of each evaluation index of Sigmoid kernel function are low.

The identification results are shown in Figure 4. According to the accuracy (Acc), precision (Pre), recall (Re), specificity (Sp), G-mean (G), and MCC, we find that the value of each evaluation index of sigmoid kernel function is very low, so the identification effect of the model is the worse, which cannot meet the needs of accurate identification. The values of each evaluation index of RBF kernel function are high, especially the values of accuracy, G-mean, and MCC are significantly greater than the corresponding values of other kernel functions. Therefore, the radial basis function (RBF) is selected as the kernel function of SVC.

Because the DBP is the feature with the highest importance score, it is the most important feature. Therefore, the subset composed of DBP is used as the first feature subset of the new feature ranking. Based on the importance ranking, new features with higher importance than the previous ones are continuously added and expanded into feature subsets with a different number of features. There are 12 different feature combinations until all 12 individual features are selected. The training set of different feature subset data are fed into the GSA-SVC algorithm, and the identification model of coal miners with abnormal physical signs is established. The test set is used to evaluate the performance of GSA-SVC model. The output of the model is the category of the predictive coal miners, accuracy, precision, recall, specificity, G-mean, and MCC of the test set. The final simplified feature subset is obtained according to the evaluation indexes of the test set.

The identification results of the test set of different feature subsets are shown in Table 3. When there is only one feature, the accuracy of the test set is the lowest, only 72.5%, and 22 samples are misclassified. From the first feature to the second feature, the accuracy, precision, and specificity of the test set increased rapidly, with an increase of 10, 18.42, and 28.57%, respectively, of which the increase of specificity was the most significant. When the number of features increases to six, each evaluation index of the test set reaches the maximum for the first time. The accuracy of the test set is 97.5%, and only two samples are misclassified, with the corresponding cost = 71.4777 and gamma = 12.4889. Compared with the model of six features, although the test set accuracy of the model with seven features is also 97.5%, the difference is mainly reflected in the three evaluation indexes of precision, recall, and specificity. However, the precision and specificity of the model with six features are better than that with seven features. The accuracy of the test set after the seven features decreases as a whole and finally tends to be stable. Based on the complexity of data and different evaluation indexes, the model that cannot only ensure the number of features as few as possible but also obtain the high performance is selected. (DBP, ALT, CHOL, GLU, TG, and RPP) as the result of mRMR algorithm selection. Blood pressure is the evaluating indicator of human cardiovascular function and maintains the oxygen and nutrition supply of body organs and tissues. The DBP value is above 90 mmHg, which represents hypertension. Abnormal blood pressure will damage the blood vessels of the human body and damage different organs such as heart, eyes, brain, and kidney. The injury of hepatocytes will release ALT into the blood and cause the increase in ALT content. Vigorous exercise, long-term and heavy drinking, eating habits, irregular life, and rest may all cause the increase in ALT content. CHOL contains all cholesterol in the human body. CHOL is produced and integrated by the liver. Abnormal CHOL will cause dyslipidemia, which will lead to angina pectoris, coronary heart disease, myocardial infarction, cerebral infarction, and other diseases. GLU is an indicator of blood glucose, abnormal GLU can lead to diabetes, human nervous system, and circulatory system diseases. TG is a blood lipid evaluation index. Too high content of TG will produce more fat, which not only increases the viscosity of blood but also causes blood vessel blockage and hypoxia in the brain and myocardium. RPP refers to the product of heart rate and systolic blood pressure. It is a sensitive reliability index of myocardial oxygen consumption. Abnormal RPP usually causes diseases such as stroke and heart failure. According to the screened physical signs, it can be seen that the abnormal physical signs of mine workers are mainly concentrated in the problem of cardiac function, liver function, blood lipid, and cardiac function.

**TABLE 3:** The identification results of the test set of different feature subsets.

Feature Subset	Number	Cost	Gamma	Acc/%	Pre/%	Re/%	Sp/%	G	MCC
{x_6_}	1	93.4226	86.2252	72.5 (58/80)	73.47	80.00	62.86	0.77	0.44
{x_6_, x_13_}	2	4.3976	49.7381	82.5 (66/80)	91.89	75.56	91.43	0.83	0.67
{x_6_, x_13_, x_15_}	3	15.0194	13.3854	87.5 (70/80)	94.87	82.22	94.29	0.88	0.76
{x_6_, x_13_, x_15_, x_16_}	4	17.9261	7.5884	87.5 (70/80)	94.87	82.22	94.29	0.88	0.76
{x_6_, x_13_, x_15_, x_16_, x_14_}	5	67.081	14.4606	93.75 (75/80)	97.62	91.11	97.14	0.94	0.88
{x_6_, x_13_, x_15_, x_16_, x_14_, x_9_}	6	71.4777	12.4889	97.5 (78/80)	97.78	97.78	97.14	0.98	0.95
{x_6_, x_13_, x_15_, x_16_, x_14_, x_9_, x_11_}	7	1.7972	42.3299	97.5 (78/80)	95.74	100	94.29	0.98	0.95
{x_6_, x_13_, x_15_, x_16_, x_14_, x_9_, x_11_, x_1_}	8	34.5154	3.3239	96.25 (77/80)	100	93.33	100	0.97	0.93
{x_6_, x_13_, x_15_, x_16_, x_14_, x_9_, x_11_, x_1_, x_8_}	9	55.8913	2.5494	95 (76/80)	97.67	93.33	97.14	0.95	0.90
{x_6_, x_13_, x_15_, x_16_, x_14_, x_9_, x_11_, x_1_, x_8_, x_24_}	10	64.1792	1.7058	95 (76/80)	95.56	95.56	94.29	0.96	0.90
{x_6_, x_13_, x_15_, x_16_, x_14_, x_9_, x_11_, x_1_, x_8_, x_24_, x_25_}	11	54.321	1.4341	93.75 (75/80)	95.45	93.33	94.29	0.94	0.87
{x_6_, x_13_, x_15_, x_16_, x_14_, x_9_, x_11_, x_1_, x_8_, x_24_, x_25_, x_3_}	12	63.1411	0.5812	93.75 (75/80)	95.45	93.33	94.29	0.94	0.87

**FIGURE 5:**
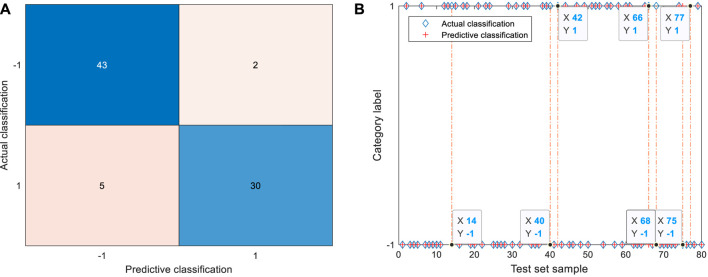
Identification results of 25 physical signs by GSA-SVC. **(A)** Confusion matrix of test set. The TP is 43, TN is 30, FP is 5, and FN is 2; **(B)** Identification results of test set. The 14th, 40th, 42nd, 66th, and 68th samples were misclassified as abnormal coal miners in the test set. The 75th and 77th samples were misclassified as normal coal miners in the test set.

The identification results of GSA-SVC of the raw 25 physical signs are shown in Figure 5. The label “−1” represents the diagnostic result indicates further clinical examination is required, and the label “1” represents the diagnostic result that no abnormality is found in the currently tested items. In Figure 5B, the blue diamond is the actual classification of input samples, and the red plus sign is the result of the predictive classification of the model. If the plus sign can fill in the diamond, it means that samples are correctly identified. The orange dotted line corresponds to the misclassified sample. The color depth of different regions of the confusion matrix reflects the number of samples corresponding to the region. The darker the color, the larger the number of samples, otherwise the less the number of samples.

According to the confusion matrix and identification results of the test set, 43 coal miners with abnormal physical signs and 30 coal miners with normal physical signs were identified correctly. The five coal miners with normal physical signs in the test set were wrongly identified as coal miners with abnormal signs. These five coal miners belong to high-risk groups, and they are more likely to have physical abnormalities in the future, which should be paid attention to. The 14th, 40th, 42nd, 66th, and 68th samples were misclassified, corresponding to the 116th, 310th, 268th, 295th, and 33rd coal miners of the physical signs data set, respectively. Two coal miners with abnormal physical signs in the test set were incorrectly identified as normal coal miners. These two coal miners belong to the missed diagnosis population, and the coal miners with abnormal physical signs were not successfully identified. The 75th and 77th samples were misclassified, corresponding to the 176th and 119th coal miners in the physical signs data set, respectively. Among the identification results of raw 25 physical signs, the misclassification results occurred most frequently in the two departments of the excavation and coal preparation plant, and there was no misclassification in the electromechanical department.

**FIGURE 6:**
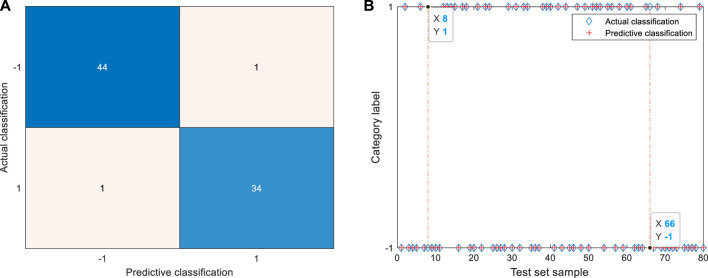
Identification results of six physical signs by GSA-SVC. **(A)** Confusion matrix of test set. The TP is 44, TN is 34, FP is 1, and FN is 1; **(B)** Identification results of test set. The 66th samples were misclassified as abnormal coal miners in the test set. The eighth samples were misclassified as normal coal miners in the test set.

The raw data are simplified to the greatest extent by EN and mRMR. The identification results of GSA-SVC of six physical signs are shown in Figure 6. According to the confusion matrix and identification results of the test set, 44 coal miners with abnormal physical signs and 34 coal miners with normal physical signs were identified correctly. One coal miner with normal physical signs was wrongly identified as a coal miner with abnormal physical signs, and the 66th sample in the test set was wrongly identified, corresponding to the 295th coal miner in the data set. One coal miner with abnormal physical signs in the test set is wrongly identified as a coal miner with normal physical signs, and the eighth sample is wrongly identified, corresponding to the 115th coal miner in the data set. The identification result of the model based on the features of raw data simplified by EN and mRMR is better than that of the raw data based on the model. The number of errors in identification has been reduced by five, which is mainly reflected by the number of real normal samples is four less than that of errors misidentified as abnormal samples.

### Comparison With Other Classifiers

To verify the reliability of the method proposed for the identification of abnormal physical signs, we compare the algorithms proposed in different stages. EN and mRMR are separately employed to select the features of the raw physical signs data, and then the selected features are used as the input of the unoptimized SVC and GSA-SVC algorithms to establish the identification model of abnormal physical signs. In the libsvm-mat-3.1 toolkit, the default value of the penalty coefficient cost is set to one, and the default value of the kernel function parameter gamma is set to the reciprocal of the feature number. Then, the feature selection method of EN combined with mRMR is used to simplify the raw data and establish the identification model of abnormal physical signs. Finally, the performance of the model is evaluated by comparing the identification accuracy and the number of features of the test set with different processing methods.

The identification results of different processing methods are shown in Table 4. The number of features and evaluation indexes of different methods is different in varying degrees. The accuracy and other evaluation indexes of the test set of the GSA-SVC model based on the raw 25 physical signs are significantly higher than those of the SVC model, which shows that the GSA is based on the swarm intelligence algorithm can greatly improve the identification accuracy of the SVC model. The accuracy of the test set of the SVC model with different methods is less than 90%, which shows that the unoptimized SVC model cannot achieve the accurate and efficient identification of abnormal physical signs. When the classifier is SVC, the test set obtained by using a single EN algorithm to process the raw data has the lowest accuracy and more features are deleted. The combination of EN and mRMR is used to process the raw data, the accuracy of the test set is high, and the number of deleted features is also small. The accuracy of the test set of the GSA-SVC model built by different methods is more than 90%. Compared with the EN algorithm, the model processed by mRMR has realized a better identification result. The value of the evaluation indexes of the test set obtained by the method proposed in this paper is larger than those of other processing methods in different stages, and the prediction effect is better. At the same time, the number of corresponding features is the least. Compared to the raw data and the data processed by single EN, the number of features is reduced by 76 and 50%, respectively.

**TABLE 4:** Identification results of different processing methods.

Method	Number	Cost	Gamma	Acc/%	Pre/%	Re/%	Sp/%	G	MCC
SVC	25	1	0.04	78.75 (63/80)	79.17	84.44	71.43	0.82	0.57
GSA-SVC	25	48.8545	0.5978	91.25 (73/80)	89.58	95.56	85.71	0.93	0.82
EN + SVC	12	1	0.0833	77.50 (62/80)	80	80	74.29	0.8	0.54
EN + GSA-SVC	12	63.1411	0.5812	93.75 (75/80)	95.45	93.33	94.29	0.94	0.87
mRMR + SVC	8	1	0.125	85.00 (68/80)	88.37	84.44	85.71	0.86	0.70
mRMR + GSA-SVC	12	25.8714	2.5898	96.25 (77/80)	95.65	97.78	94.29	0.97	0.92
EN + mRMR + SVC	7	1	0.1429	88.75 (71/80)	87.50	93.33	82.86	0.90	0.77
Proposed	6	71.4777	12.4889	97.50 (78/80)	97.78	97.78	97.14	0.98	0.95

We combine the processing method of EN and mRMR with SVC under different intelligent optimization algorithms to evaluate the performance of the model to verify its novelty and superiority of the proposed model. The compared intelligent optimization algorithms include the grid-search (GS), genetic algorithm (GA), and particle swarm optimization (PSO). The initial population number is set to 20, the maximum number of iterations is set to 100, the search interval of penalty coefficient cost and kernel function parameter gamma are respectively set to (0,100), and the data are normalized to (0,1) to ensure the unity of initial conditions.

The comparison results of different intelligent optimization algorithms are shown in Table 5. The SVC model with optimized parameters all achieves the best identification effect at six features. The test set accuracy of SVC optimized by GS, GA, and PSO is 96.25%, and other evaluation indexes can also reflect the same identification effect. The difference between the first three optimization strategies is mainly reflected in the evaluation index of the training set. The training set of GS and GSA has the highest accuracy. The model based on GSA can achieve a better identification result by comprehensively comparing the evaluation indexes of the training set and the test set.

**TABLE 5:** Comparison results of different intelligent optimization algorithms.

Optimization algorithm	Number	Training set						Test set					
		Acc/%	Pre/%	Re%	Sp/%	G	MCC	Acc/%	Pre/%	Re/%	Sp/%	G	MCC
GS- SVC	6	99.17	99.25	99.25	99.06	0.99	0.98	96.25	95.65	97.78	94.29	0.97	0.92
GA- SVC	6	97.08	95.68	99.25	94.34	0.97	0.94	96.25	95.65	97.78	94.29	0.97	0.92
PSO- SVC	6	97.92	97.08	99.25	96.23	0.98	0.96	96.25	95.65	97.78	94.29	0.97	0.92
GSA-SVC	6	99.17	99.25	99.25	99.06	0.99	0.98	97.50	97.78	97.78	97.14	0.98	0.95

The decision tree (DT), k-nearest neighbors (KNN), random forest (RF), naive bayesian (NB), and extreme learning machine (ELM) are commonly used machine learning algorithms, and they belong to supervised learning. First of all, the training set of 25 feature data are fed into EN algorithms, and variables with zero coefficient are deleted. Then, the remaining variables are used as inputs to mRMR and different feature subsets are generated. Finally, the training set of different feature subset data are fed into the DT, KNN, RF, NB, and ELM algorithms. The identification model of coal miners with abnormal physical signs is established. The test set is used to evaluate the performance of the above models. The output of the model is the category of the predictive coal miners, accuracy, precision, recall, specificity, G-mean, and MCC of the training set and test set. The number of features is obtained according to evaluation indexes. Multiple evaluation indexes of the training set and test set of different classifiers are compared to evaluate the identification performance of different classifiers. The comparison results are shown in Table 6. The accuracy and other evaluation indexes of the training set and test set of the KNN are low. Although the accuracy of the test set of the RF, NB, and ELM is the same, which indicates that they have similar identification performance, the accuracy of the training set of the NB is the lowest. The identification effect of the test set of the DT is closest to the proposed classifier, but most of the evaluation indexes such as the accuracy of the training set are worse than the proposed classifier, and the number of reduced features is more. The KNN, RF, and the proposed classifier finally reduced the number of features to six. The number of features selected by NB is the most, which is about twice as many.

**TABLE 6:** Comparison results of different classifiers.

Classifier	Number	Training set						Test set					
		Acc/%	Pre/%	Re%	Sp/%	G	MCC	Acc/%	Pre/%	Re/%	Sp/%	G	MCC
DT	8	98.33	97.79	99.25	97.17	0.99	0.97	97.50	100.00	95.56	100.00	0.98	0.95
KNN	6	90.42	95.87	86.57	95.28	0.91	0.81	93.75	97.62	91.11	97.14	0.94	0.88
RF	6	100.00	100.00	100.00	100.00	1.00	1.00	96.25	95.65	97.78	94.29	0.97	0.92
NB	11	90.00	95.08	86.57	94.34	0.91	0.80	96.25	97.73	95.56	97.14	0.97	0.92
ELM	7	93.33	93.27	91.51	94.78	0.92	0.86	96.25	94.44	97.14	95.56	0.96	0.92
Proposed	6	99.17	99.25	99.25	99.06	0.99	0.98	97.50	97.78	97.78	97.14	0.98	0.95

**FIGURE 7:**
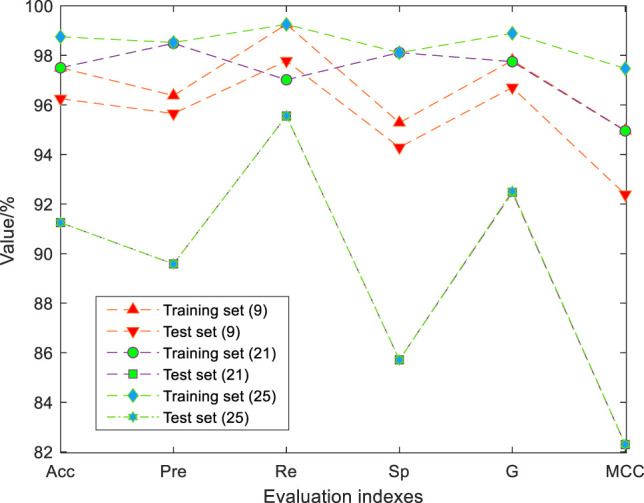
Comparison of results after simplifying features. The values of each evaluation index of test set (9) are high. The accuracy, precision, recall, specificity, G-mean, and MCC of the test set were 96.25%, 95.65%, 97.78%, 94.29%, 0.97, and 0.92. The values of each evaluation index of test set (21) are low. The accuracy, precision, recall, specificity, G-mean, and MCC of the test set were 91.25%, 89.58%, 95.56%, 85.71%, 0.93, and 0.82.

It needs to be verified that the new features generated by feature construction can improve the identification performance of the model, rather than adding some useless features to increase the complexity of algorithm operation. We combined EN-mRMR with GSA-SVC to simplify the 21 physical signs data before feature construction. The simplified results are shown in Figure 7. When the number of physical signs is nine, the GSA-SVC model achieves the best identification results. The subset of nine features is (DBP, RPP, CHOL, GLU, TG, WGT, SBP, HR, AGE, FEV1%, FEV1/FVC%, and TWA). The accuracy, G-mean, and MCC of the training set of the identification model with nine physical signs and 21 physical signs data are the same, and the values are 97.5, 98, and 95%, respectively. There is no significant difference in the identification effect of the training set. The evaluation indexes of the test set are significantly better than that of 21 physical signs. Compared to the evaluation indexes of the model of the raw data with 21 physical signs, the EN-mRMR method can also improve the identification performance of the model. The performance indexes of the test set with 21 physical signs and the test set with 25 physical signs after feature construction are the same. The Identification performance of the model with the training set of 25 physical signs is better.

The data of 21 physical signs are also used as the input of the DT, KNN, RF, NB, and ELM and combined with EN-mRMR to simplify the feature number of the data. First of all, the training set of 21 feature data are fed into EN algorithms, and variables with zero coefficient are deleted. Then, the remaining variables are used as inputs to mRMR and different feature subsets are generated. Finally, the training set of different feature subset data are fed into the DT, KNN, RF, NB, and ELM algorithms. The identification model of coal miners with abnormal physical signs is established. The test set is used to evaluate the performance of the above models. The output of the model is the category of the predictive coal miners, accuracy, precision, recall, specificity, G-mean, and MCC of the training set and test set. The number of features is obtained according to evaluation indexes. Multiple evaluation indexes of the training set and test set are compared to evaluate the identification performance of different classifiers. The comparison results are shown in Table 7. The test set of the DT, KNN, RF, and ELM has a similar identification performance. The number of features reduced by DT, KNN, and RF is the same. Although the evaluation indexes of the test set are higher than those of other classifiers, the accuracy, recall, and MCC of the training set are very low, and the number of selected features is the largest. Overall, the EN-mRMR can still achieve better identification performance by simplifying the data without feature construction and combining the nine physical signs obtained with the GSA-SVC classifier.

**TABLE 7:** Comparison results of different classifiers.

Classifier	Numbers	Training set						Test set					
		Acc/%	Pre/%	Re/%	Sp/%	G	MCC	Acc/%	Pr/%	Re/%	Sp/%	G	MCC
DT	7	99.58	100.00	99.25	100.00	1.00	0.99	95.00	100.00	91.11	100.00	0.95	0.90
KNN	7	91.25	90.65	94.03	87.74	0.92	0.82	95.00	95.56	95.56	94.29	0.96	0.90
RF	7	100.00	100.00	100.00	100.00	1.00	1.00	95.00	95.56	95.56	94.29	0.96	0.90
NB	11	89.58	96.58	84.33	96.23	0.90	0.80	97.50	100.00	95.56	100.00	0.98	0.95
ELM	8	93.75	93.33	92.45	94.78	0.93	0.87	95.00	94.29	94.29	95.56	0.94	0.90
SVC	8	83.33	83.57	87.31	78.30	0.85	0.66	87.50	85.71	93.33	80.00	0.89	0.75
Proposed	9	97.50	96.38	99.25	95.28	0.98	0.95	96.25	95.65	97.78	94.29	0.97	0.92

Referring to Tables 3, 6, 7; Figure 7, it can be found that feature construction improves the identification performance of GSA-SVC and other classifiers at the same time. The EN-mRMR combined with different classifiers is used to simplify the data after feature construction, and the number of features after is less than the raw data before feature construction. In particular, the method of EN-mRMR combines with GSA-SVC for data with feature construction is better than the data without feature construction, and increases the evaluation indexes for the training set and test set.

## Discussion and Conclusion

In this paper, we put forward a new strategy for identification of coal miners with abnormal physical signs based on EN-mRMR. Firstly, we constructed some features of related tasks through feature construction technics. Then the EN was used to delete the redundant physical parameters. Finally, we combined the mRMR with the GSA-SVC algorithm to establish the identification model of coal miners with abnormal physical signs. The features of the preliminarily selected data of physical signs were simplified, and the most important feature subset was obtained. To verify the reliability of the proposed strategy, we compared the identification performance of the proposed algorithms in different stages. We compared the identification performance of different classifiers to verify the novelty and superiority of the proposed model. We also compared the model after feature construction with the model before.

The analysis of the experimental process and the evaluation of the final results show that: 1) The single feature selection method of EN or mRMR can delete redundant physical signs, select useful features, reduce the complexity of data, and avoid the interference and indifferent impact of redundant data on the identification performance of the model. The number of physical signs selected by EN or mRMR is 48% of the number of all physical signs. 2) Compared to a single feature selection algorithm, the proposed strategy in this paper can reduce the number of features of the data to the greatest extent, and those fewer features are sufficient to reflect the key information of the raw data. The number of physical signs selected by EN-mRMR is only 24% of the number of all physical signs. 3) The GSA is employed to optimize the parameters of SVC. The established identification model not only has a higher identification accuracy but also avoids the over-fitting of the model to a certain extent. The accuracy of the training set and test set are 99.17 and 97.50%, respectively. 4) The data after feature construction are selected by EN-mRMR combined with different classifiers. The number of feature reduction and the identification performance of the model is improved to varying degrees. 5) This experiment verifies the feasibility of EN-mRMR combined with GSA-SVC for the identification of coal miners with abnormal physical signs. The proposed strategy in this paper improves the modeling efficiency and model performance with fewer features and realizes the accurate identification of abnormal physical signs.

Despite the achievement of some research results, there are some limitations in this study. Due to the limitation of sample data and diagnosis results, the diagnosis results of normal and abnormal physical signs are identified based on a small sample size in this paper, which is primarily for early screening and early warning for the detection of coal miners’ occupational and suspected occupational diseases. Therefore, we plan to collect more data samples of different kinds of diseases on the conditions permitted to identify and evaluate different types of occupational diseases.

## Data Availability

The data in this study can be obtained from the corresponding author. Requests to access the datasets should be directed to Kai Bian, 422088134@qq.com.
